# Supplementary material for: Inhibition of Renal Stellate Cell Activation Reduces Renal Fibrosis
Source: Biomedicines. 2020 Oct 19;8(10):431. doi: 10.3390/biomedicines8100431 (PMC7603238; doi:10.3390/biomedicines8100431)
Supplement: Supplementary file 1 [file biomedicines-08-00431-s001.pdf]

biomedicines
MDPI

**Supplemental Table 1.** Primers used in this study.

| Gene | | 5' to 3' |
|---|---|---|
| *mβ-actin* | F | GGACTCCTATGTGGGTGACG |
| | R | CTTCTCCATGTCGTCCCAGT |
| *mCollagen I* | F | CCAAAGGTGCTGATGGTTCT |
| | R | ACCAGCTTCACCCTTGTCAC |
| *mMCP1* | F | CTGGATCGGAACCAAATGAG |
| | R | CGGGTCAACTTCACATTCAA |
| *mPAI-1* | F | TCCTCATCCTGCCTAAGTTCTC |
| | R | GTGCCGCTCTCGTTTACCTC |
| *mSTRA6* | F | TGCTGGACTCTGGAGATG |
| | R | GTGATCACCTGCCCATC |
| *mTGFβ1* | F | AGCCCGAAGCGGACTACTAT |
| | R | CTGTGTGAGATGTCTTTGGTTTTC |
| *rAlbumin* | F | CGGTACCGGCACAATGAAGTGGGTAA |
| | R | GGTCTAGATTAGGCTAAGGCTTCTTTG |
| *rAggf1* | F | AAGGCCGGAAGATGTTGGAG |
| | R | CTCTCGTGCTTTGTCCCAGT |
| *rα-SMA* | F | TATCTGGGAAGGGCAGCAAA |
| | R | CCAGGGAAGAAGAGGAAGCA |
| *rCEBPα* | F | AAGATGCGCAACCTGGAGAC |
| | R | CCTTCTTCTGCAGCCGCTC |
| *rCollagen I* | F | GGAGAGTACTGGATCGAC |
| | R | CTGACCTGTCTCCATGTT |
| *rCrbp1* | F | CACTACCCACCCATTTCGCT |
| | R | GGGTGGAGGGGTAAGAAAGC |
| *rGAPDH* | F | GGTGGTCTCCTCTGACTTCAACA |
| | R | GTTGCTGTAGCCAAATTCGTTGT |
| *rPPARγ* | F | CACAATGCCATCAGGTTTGG |
| | R | GCTGGTCGATATCACTGGAGATG |
| *rPDGF-β* | F | TGGAGTCGAGTCGGAAAGCT |
| | R | GAAGTTGGCATTGGTGCGAT |
| *rNG2* | F | GTTTACCCTCACCACTCGGA |
| | R | TAAAGTTGCCACGCTTGTCC |

**Supplemental Table 2.** Primary antibodies used in this study.

| Method | Primary antibody | Company and Country |
|---|---|---|
| Immunohistochemistry | α-SMA | Dako, Santa Clara, CA, USA |
| | CD31 | Cell Signaling Technology, Beverly, MA, USA |
| | Collagen I | Abcam, Cambridge, MA, USA |
| | Collagen IV | Abcam, Cambridge, MA, USA |
| | Cygb/STAP | Generous gift from Dr. Norifumi Kawada |
| | Desmin | Dako, Carpinteria, CA, USA |
| | Fibronectin | Abcam, Cambridge, MA, USA |
| | F4/80 | Serotec, Kidlington, Oxford, UK |
| | His-tag | Bioss, Woburn, MA, USA |
| | PAI-1 | American Diagnostica, Stamford, CT, USA |
| | PDGFR-β | Enzo, Farmingdale, NY, USA |
| | STRA6 | Bioss, Woburn, MA, USA |
| | TGF-β1 | Santa Cruz Biotechnology, Santa Cruz, CA, USA |
| Western blotting | Albumin | Affinity Bioreagents, Rockford, Illinois, USA |
| | α-SMA | Sigma-Aldrich, St. Louis, MO, USA |
| | α-tubulin | Cell Signaling Technology, Beverly, MA, USA |
| | β-actin | Sigma-Aldrich, St. Louis, MO, USA |
| | E-cadherin | Abcam, Cambridge, MA, USA |
| | FSP-1 | Abcam, Cambridge, MA, USA |
| | LRAT | IBL, Gunma, Japan |

**Supplemental Figure 1.** Schematic diagram of R-III, a retinol-binding protein (RBP) - albumin domain III fusion protein, in comparison with full-length albumin and RBP. The numbers indicate amino acids.

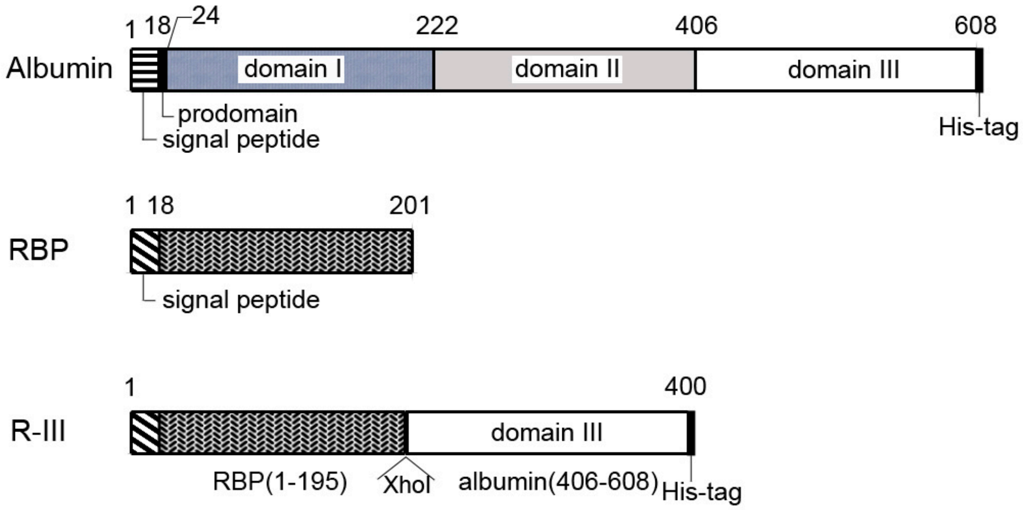

**Supplemental Figure 2.** Differential interference contrast images of oil red O staining for renal stellate cells on day 3 after seeding.

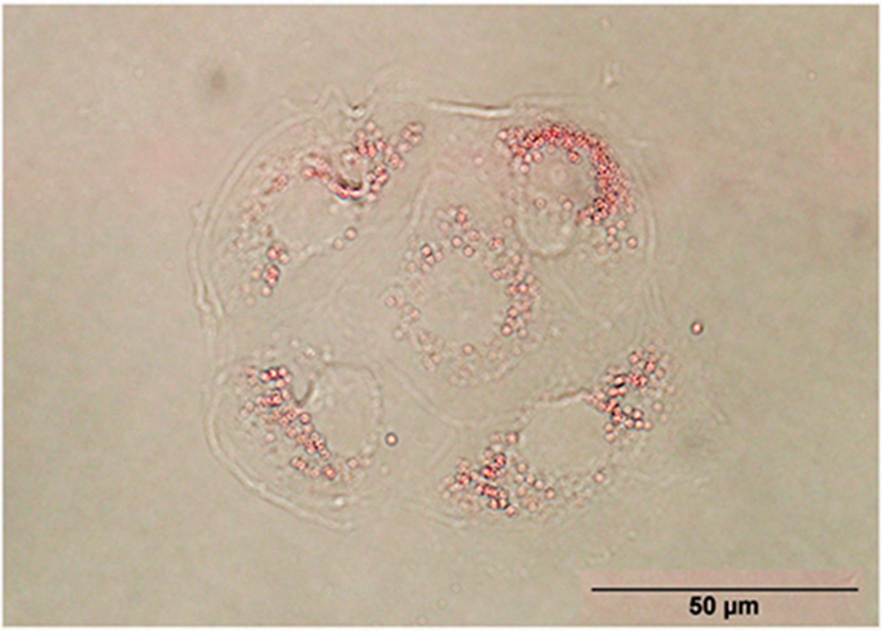

**Supplemental Figure 3.** Renal stellate cells after passage 2 were treated with bovine serum albumin (BSA; 0.5 μM) or R-III (0.5 μM) for 20 h, and α-smooth muscle actin (α-SMA) expression was analyzed by real-time PCR. The data represent the means ± SD for three independent experiments. *P*-value was estimated using paired *t*-test (compared with the untreated cells). $^{**}P < 0.01$. ns = not significant.

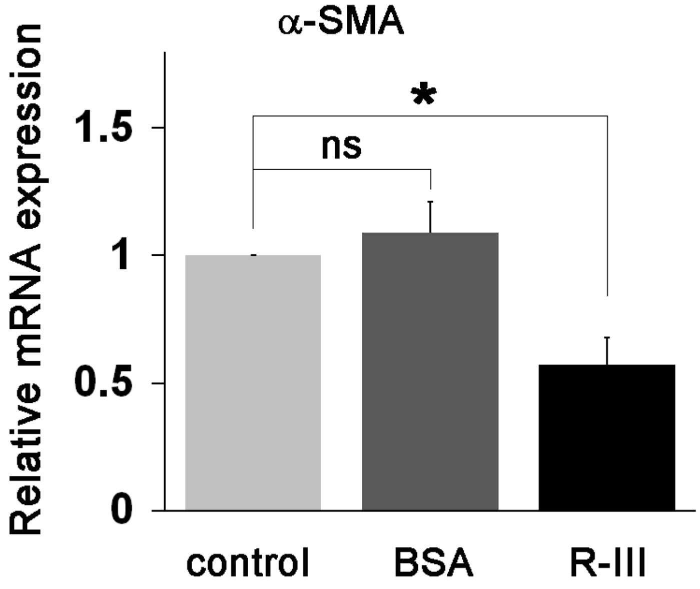

**Supplemental Figure 4.** Uncropped full-length blot images.

Figure 2D

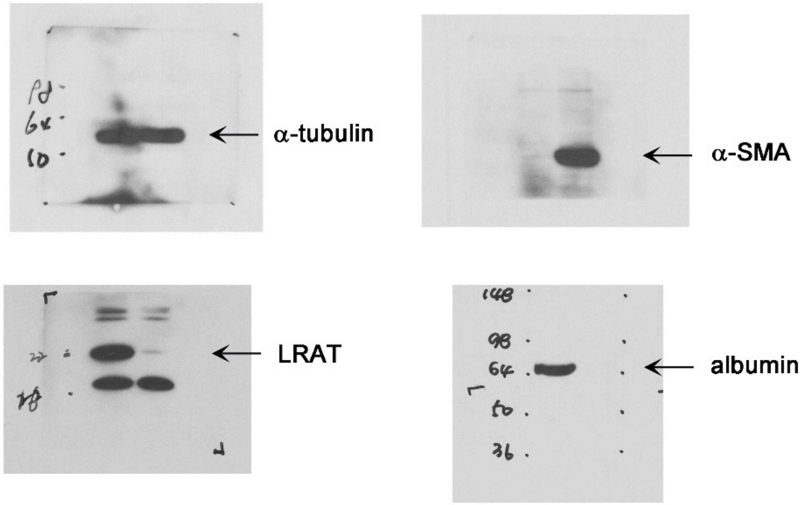

**Supplemental Figure 5.** Schedules for developing the murine kidney injury model using unilateral ureteral obstruction (UUO). Male C57BL/6 mice were subjected to UUO, and were intravenously administered saline or R-III (30 μg) dissolved in saline daily at 8–14 days after UUO (*hatched box, black arrows*).

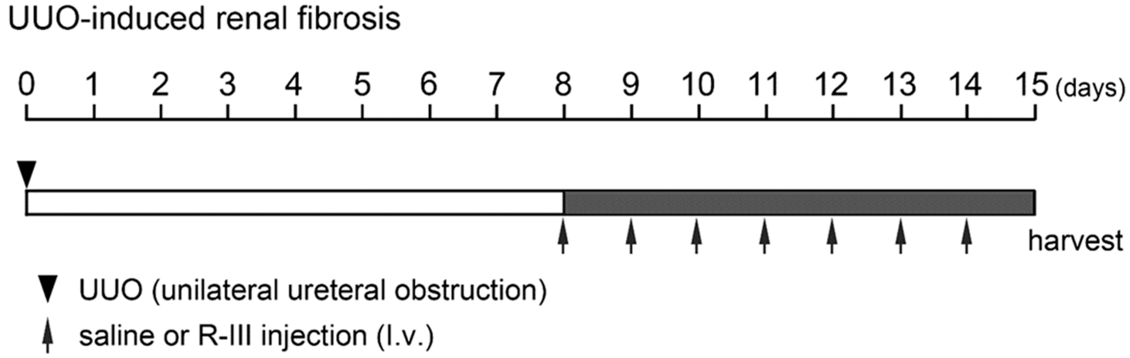

**Supplemental Figure 6.** Effects of R-III on epithelial-mesenchymal transition (EMT) *in vitro* and *in vivo*. (A) NRK-52E cells were treated with TGF-β1 (50 ng/ml) for 5 days in the presence or absence of R-III (0.5 μM) and subjected to immunofluorescence using antibodies against E-cadherin or α-SMA. (B) Quantitative assessment of the intensity of α-SMA staining. The data represent the means ± SD for five independent experiments. *P*-value was determined using paired *t*-test. *$P < 0.05$ vs. control, #$p < 0.05$ vs. TGF-β1-treated cells. (C) Tissue lysates were prepared from the kidneys of sham-, UUO-, and UUO+R-III-treated mice and analyzed by western blotting for the expression of EMT markers. (D) Densitometric analysis of the bands on the western blot. Data are expressed as the means ± SD (*P*-value; Kruskal–Wallis test, followed by DSCF multiple comparison test). **$P < 0.01$ vs. Sham, #$p < 0.05$ vs. UUO treatment.

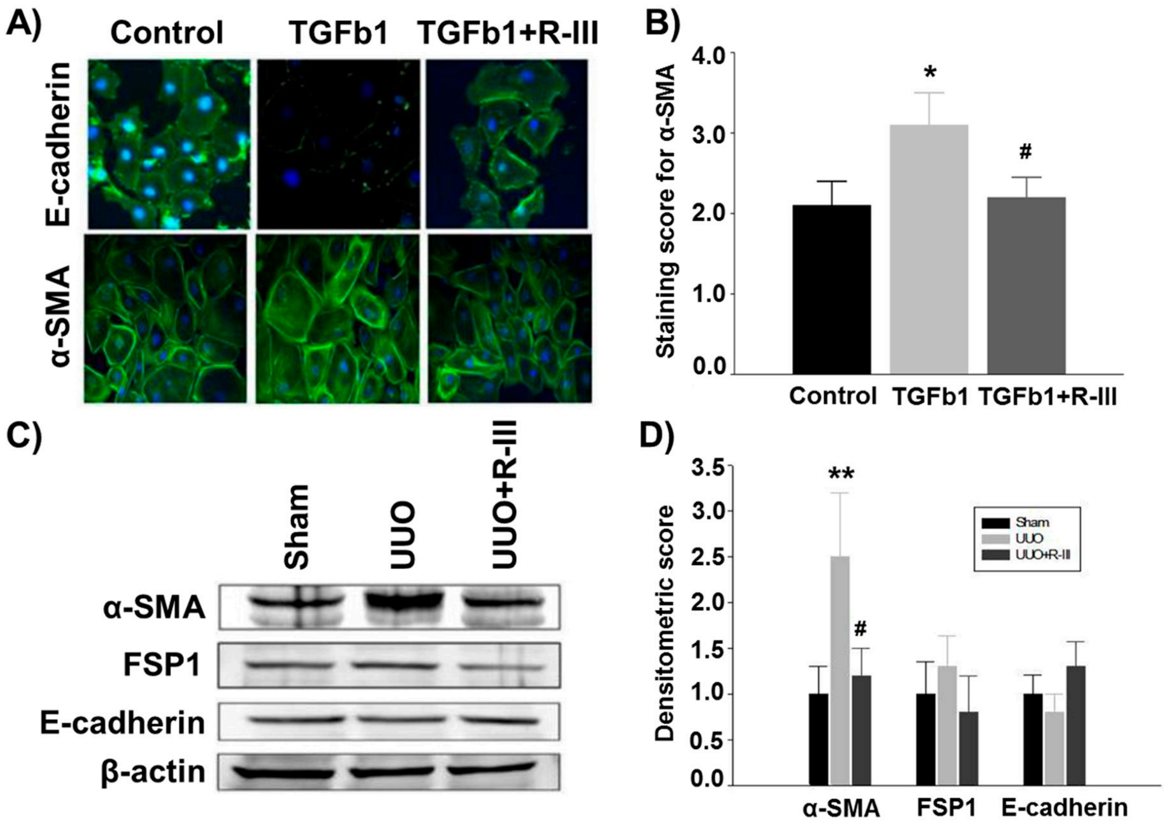

## Supplemental Methods

### Epithelial-mesenchymal transition (EMT)

NRK-52E cells were seeded on 0.1% gelatin-coated coverslips in a 24-well plate and grown in Dulbecco`s Modified Eagle Media (DMEM) containing 5% fetal bovine serum (FBS). Subconfluent cells were starved for 24 h by incubating with DMEM containing 1% FBS, and then cultured for five days in the presence of recombinant mouse TGF-β1 (50 ng/ml, Cell Signaling Technology, Danvers, MA, USA) to induce EMT. To examine the effects of R-III, cells were treated with R-III (0.5 μM) by adding it to the culture medium during the last 24 h of TGF-β1 treatment.
